# Strengthening health systems to tackle oral diseases in Africa: Africa centers for disease control and prevention’s role

**DOI:** 10.3389/fpubh.2025.1539805

**Published:** 2025-01-23

**Authors:** Moréniké Oluwátóyìn Foláyan, Adeyinka Ganiyat Ishola, Ahmed Bhayat, Maha El Tantawi, Nicaise Ndembi

**Affiliations:** ^1^The Africa Oral Health Network (AFRONE), Alexandria University, Alexandria, Egypt; ^2^Department of Child Dental Health, Obafemi Awolowo University, Ife, Nigeria; ^3^Department of Nursing, University of Ibadan, Ibadan, Nigeria; ^4^Department of Community Dentistry, University of Pretoria, Pretoria, South Africa; ^5^Department of Pediatric Dentistry and Dental Public Health, Faculty of Dentistry, Alexandria University, Alexandria, Egypt; ^6^Africa Centres for Disease Control and Prevention, Addis Ababa, Ethiopia

**Keywords:** equity, integrated health systems, workforce development, health security’ health systems strengthening, systemic inequities, health workforce retention, universal health coverage

## Abstract

Oral diseases remain a significant public health challenge in Africa. Despite their prevalence, oral health is often overlooked in national health agendas and universal health coverage frameworks. This manuscript explores the crucial role of the Africa Centers for Disease Control and Prevention (Africa CDC) in strengthening health systems to address the growing oral health problem in Africa. A rapid review of the literature was conducted in databases like PubMed and ScienceDirect identified 18 relevant studies focusing on workforce size, retention, distribution, patient access, and system outcomes. The analysis revealed severe workforce shortages, uneven distribution, and systemic neglect, particularly in rural areas. Promising interventions included dental education programs, task-shifting to mid-level workers, and mobile dental units. Key recommendations emphasize integrating oral health into national policies, addressing inequities, expanding training, and improving infrastructure and workforce retention through incentives. By leveraging its position and resources, the Africa CDC can take strategic actions to strengthen surveillance and data-driven policy development, provide technical assistance to Africa Union Member States for health system integration, support capacity building for oral health workforce development, promote preventive and community-based oral health interventions, facilitate cross-border collaboration and knowledge sharing, mobilize resources and funding for oral health programs, and support the local production of oral health products. These all aligns with the institution’s new public health order for Africa’s health security and one of the institutions’ 2023–2027 goals.

## Introduction

Oral diseases such as dental caries, periodontal diseases, oral cancers, HIV-related oral lesions, oro-dental and facial trauma, cleft lip and palate, and noma are widespread across Africa (1–3), significantly contributing to morbidity (4), mortality (5), and reducing quality of life (6). In 2019, an estimated 28.5% of people over 5 years old in the World Health Organization (WHO) African Region had untreated caries in their permanent teeth, while 38.6% of children aged 1–9 years had untreated caries in primary teeth, and 22.8% of individuals aged 15 and older suffered from severe periodontal disease. The highest prevalence of severe periodontitis worldwide occurs in the WHO African region (7). In addition, in 2020, the incidence of oral cancer (lip and oral cavity cancers) ranged from 0.4 to 6.6 per 100,000 people, and 30–80% of people living with HIV experienced oral lesions (8). Oral disease affects an estimated 480 million people (43.7%) in the WHO African Region in 2019 (8) and this burden is growing due to the population growth in the region (9).

Oral diseases disproportionately affect vulnerable and disadvantaged populations including people on low incomes, people living with disabilities, older people living alone or in care homes, and those living in remote and rural communities (10). In addition, women and children (11), and adolescents (12) are further disproportionately affected due to additional barriers they have to face to access preventive and curative oral health services (13). For women, social, cultural, and economic factors often limit access to healthcare services, as they may prioritize family needs over their own or face restrictions on mobility and financial independence (14–16). Children and adolescents, encounter challenges due to limited availability of pediatric oral health services (17), lack of parental awareness (18), and, in many cases, absence of school-based oral health programs (19). Adolescents, particularly, may lack autonomy to seek care independently (20) and often fall through the gaps in health systems that do not cater specifically to their developmental needs. These compounded barriers result in delayed or forgone oral healthcare, contributing to a cycle of poor oral health outcomes and widening health disparities among these vulnerable groups.

Sadly, many African countries still neglect oral health in their national health strategies (21), although the WHO (22) and other stakeholders (23) have recognized the importance of integrating oral health into health policies (24). The integration of oral health into non-communicable diseases (NCDs) programs is key because oral diseases share key modifiable risk factors—such as high sugar intake, tobacco use, harmful alcohol consumption (25) and consumption of ultra-processed meals (26)—with major NCDs like cardiovascular disease, cancer, diabetes, and chronic respiratory diseases (27). The burden of NCDs in the region is growing (28) just like the burden of oral diseases (29) making the disconnect between oral and general health services a challenge.

The African regional oral health strategy seeks to integrate oral health within NCDs management, aligning with the global push toward universal health coverage (UHC) and the WHO’s recent Oral Health Strategy (30, 31). These are political commitments to advance oral health worldwide. The Africa CDC has a pivotal role in addressing these challenges by advocating for the integration of oral health into existing health systems, promoting preventive measures, and enhancing health literacy. This is in line with the new public health order for Africa that can otherwise be tagged as the continental health system strengthening agenda (32).

While dental services remain underutilized worldwide, this challenge in Africa could be addressed through unique pathways that integrate culturally resonant practices, community-based approaches, and innovative healthcare delivery models tailored to the continent’s diverse needs. This manuscript explores strategies the Africa CDC can implement to strengthen health systems for improving oral health outcomes in Africa taking cognizance of the roles and responsibilities of the Africa CDC, its current responses to health crisis in Africa, and the oral health needs of the region.

## Status of the oral health systems in countries in Africa

A well-functioning health system relies on skilled and motivated healthcare workers, robust infrastructure, and a dependable supply of medicines and technologies. These should be supported by sufficient funding, comprehensive health strategy and evidence-based policies to ensure effectiveness (33).

A rapid review of the literature was applied for publications in English addressing oral health workforce and oral health systems strengthening in Africa. The search was conducted in EBSCO, Pubmed, ProQuest and Science Direct utilizing keywords and Medical Subject Headings (MeSH) terms related to “oral health workforce,” health system strengthening,” “dental professional shortages,” “Africa,” “recruitment,” “retention,” and “workforce strategies.” The references of the included studies were further reviewed for additional publications. Studies focusing on oral health professionals in Africa, including dentists, dental hygienists, dental therapists, and other dental healthcare providers, aimed at recruiting, training, retaining, or deploying oral health professionals. The primary outcomes assessed were workforce size, retention, distribution, patient access, and oral health outcomes. The secondary review outcomes were oral health system strengthening. Studies included were cohort studies, cross-sectional studies, and qualitative studies.

A total of 2014 articles were retrieved and 1,069 were screened following the removal of duplicates. Of these, seven article’s full texts were screened and their references were also screened for additional articles. In total, 18 articles were identified. The data on author and year of publication, study location, design and objective(s), study findings and recommendations were extracted as shown in Table 1.

**Table 1:** A Rapid review of studies reporting on oral health workforce and oral health systems strengthening in Africa.

Author and year of publication	Study location	Study design: Study objective	Study findings	Study recommendation
Aloshaiby et al. 2024 (34)	Libya	Observational study: Describe the Libyan oral health care system in terms of its structure, function, workforce, funding, reimbursement and target groups	Oral health services are integrated into medical services, primarily treatment-focused, private sector care is major provider, majority of care provided by general dental practitioners. Public sector services are limited to emergency care and extractions. Children have high prevalence of oral health problems. There is significant shortage of dental technicians and nurses and no clear policies	Urgent need to develop policies and plans to improve the oral health care system in Libya
Gallagher et al. 2023 (35)	The World Health Organization African Region (AFR): 47 countries	Descriptive analysis: review the oral health workforce comprising dentists, dental assistants and therapists, and dental prosthetic technicians in the AFR	Despite a growth in dentist density since 2010, it still remains low at 0.44 per 10,000 population. Key challenges include workforce maldistribution and the low priority given to oral health.	Urgent need to strengthen policy, health, and education systems to expand the oral health workforce using innovative workforce models to meet the needs of this region and achieve Universal Health Coverage
Yamalik et al. 2014 (36)	FDI countries including Mozambique, Morocco, Burundi, Burkina Faso, Congo, Benin	Survey: to provide a description of the oral health workforce structure, its distribution, trends, undergraduate and specialist education as well as the compliance of local regulations with oral health needs in a sample of FDI participating countries.	Mozambique, Morocco, Burundi, Burkina Faso, Congo, and Benin all face challenges in their dental healthcare systems, though to varying extents. Mozambique, has 72 dentists, lack rural dentists, and insufficient specialist care, while dental education and regulations need reform. Morocco, has a larger dental workforce (3,500 dentists) and meets specialist workforce needs but still faces uneven rural dentist distribution and regulatory issues. Burundi and Burkina Faso both suffer from low numbers of dentists and a lack of specialists and rural care, with dental education needing reform. Congo has only 71 dentists and faces significant gaps in specialist care and rural dentist availability. Benin, has 65 dentists and 400 dental graduates per year, but still faces shortages in rural dentists and specialist care, along with the need for reforms in education and regulations	Optimum or ideal oral health workforce and fair distribution of the available workforce does not seem to be achieved in many parts of the world. Further attention also needs to be dedicated to general trends that have the capacity to affect future oral health workforce
Tiwari et al. 2021 (74)	South Africa	Modeling: to determine the projected shortfall of dentists and specialists in each of the nine provinces in South Africa	In the best-case scenario, the estimated shortfall of dentists and specialists was 430 in 2018, 1,252 in 2024, and 1,885 in 2030. Under an optimistic scenario, the national shortfall increased to 733 in 2018, 1,540 in 2024, and 2,158 in 2030. In the aspirational scenario, projected shortfalls were 853 in 2018, 1,655 in 2024, and 2,267 in 2030	Access to oral health services should be ensured through the optimum supply of trained dentists and specialists and the delivery of appropriate oral health services
Bhayat and Chikte, 2018 (44)	South Africa	Descriptive analysis: to determine the demographic profile of dentists and dental specialists (DS) between 2002 and 2015	In 2015, 6,125 dentists and 481 dental specialists (DS) were registered. Younger dentists were predominantly Black and Asian women, while older dentists were mainly White males. Most DS were maxillofacial surgeons (30%), orthodontists (30%), and prosthodontists (17%). Dentist numbers grew by 2% annually, with most dentists and DS concentrated in metropolitan provinces. Over 13 years, the number of female dentists nearly doubled, and there was a sharp increase in Colored, Black, and Asian/Indian dentists and DS.	The population to dentist ratio was fairly low. The sharp increase in the number of Colored, Black and female dentists could be a result of increased admission of previously disadvantaged students to dental schools.
El Tantawi et al., 2020 (37)	Egypt	Ecological study: assessed dentist availability in Egypt including (1) changes over 20 years; (2) spatial distribution; and (3) association with supply, potential demand for care and economic conditions	Dentists per 1,000 population were randomly distributed over the country and the ratio reached 0.18 in 2014, indicating a shortage despite the increasing number of dental graduates since 1995 (667.1%). Previous dentist availability (η2 = 0.60) and increase in wages (η2 = 0.48) had the greatest impact on dentist availabilit	Egypt faces a problem of dentist shortage that has not been offset by the increase in dental graduates. Improving the economic conditions and incorporating health care into the national development plan may improve the situation
Ajao, 2018 (41)	Nigeria	Descriptive analysis: to map the current oral health workforce situation in Nigeria and explores the possibility for skill-mix and the contribution to workforce capacity that could be made by dental care professionals, including dental hygienists, therapists and nurses	Approximately 20% of Nigerian dentists practice abroad, contributing to workforce shortages. Only 20% of dentists serve rural areas, where over half of the population resides. Annually, 200 dentists, 40 hygienists, 30 therapists, and 100 nurses are trained. However, the dentist-to-population ratio is 1:44,830, far below the WHO recommendation of 1:5,000, indicating a significant demand–supply gap. Workforce challenges are compounded by migration, poor working conditions, and limited rural coverage	The current dentist workforce meets only 10% of Nigeria’s oral health needs. Expanding skill-mix roles and conducting regular national surveys are essential to address gaps and determine demand. Expanding roles for mid-level workers like hygienists and therapists could partially address this shortfall.
Monguambe and Forgay, 1998 (40)	Mozambique	Narrative textual evidence: Not available	Training programs for dental therapists and assistants were established in 1975. Previously trained Mozambican dental therapists also took additional courses and assumed leadership and principal tutor’s roles at the new training center. Regular educational seminars were held in the respective provinces to strengthen the capability of dental workers nationally.	NA
Hackley et al., 2018 (38)	Rwanda	Narrative textual evidence: To summarize the context and development of the new dental school and describes the three management tools developed by the Human Resources for Health oral health team.	The three management tools developed by the team at Rwanda’s first school of dentistry in collaboration with three American dental institutions as part of the Human Resources for Health program—a comprehensive roadmap for curriculum delivery and resource management, individual work plans for U.S. faculty tracked through biweekly flash reports, and a redesigned HRH twinning model that pairs U.S. faculty with multiple Rwandan counterparts based on shared academic interests, − enabled them to understand the successes and challenges in moving toward the planned targets and measure their progress towards the collaborative goals.	The tools have helped foster discussion around priorities and deployment of resources as well as develop strong relationships, enabling two-way exchange of knowledge, and promoting sustainability This enhanced efficiency, engagement, and collaboration impact.
Seymour et al., 2013 (39)	Rwanda	Narrative textual evidence: Not available	The ‘Human Resources for Health’ program was developed to tackle the country’s significant health care challenges by improving existing health education programs. This initiative involves a partnership to ensure that Rwanda’s health sciences education system produces a sufficient number of high-quality health care professionals, thus guaranteeing a sufficient supply for the future.	The oral health workforce training in Rwanda is expected to lead to more robust oral health data collection, a well-trained and well-retained dental profession, and vastly improved oral health and overall health for the people of Rwanda in the decades to come.
Rudolph et al., 1992 (42)	South Africa	Narrative textual evidence: Describe the evolution, development, and utilization of a purpose-built mobile dental unit during the period 1988–90.	The mobile dental unit provide comprehensive care with curative and preventive services by dental auxiliaries based using the primary oral health care approach. It also offered students the opportunity for acquiring clinical skills.	The mobile dental unit visited rural and urban communities, served several thousand patients and proved to be an effective and viable alternative oral health delivery.
Molete et al., 2016 (43)	South Africa	Economic evaluation: to undertake a cost-analysis of a school based oral health care program. Objectives: to estimate the general costs of the program, costs of oral health care per patient and the implications of providing the services at scale.	The school based oral health care program in a mobile dental unit service provided oral health screening, basic oral health treatment and referral when beyond the scope of the mobile units. The personnel costs were the highest cost drivers, followed by vehicles, equipment, and dental materials. The mobile dental unit was cost efficient and a dental therapy led service could save costs by 9.1%.	Expanding the services to a wider population of children and utilizing Dental Therapists as key personnel could improve the efficiency of mobile dental healthcare provision
Holtshousen and Smit, 2007 (75)	South Africa	Economic evaluation: To determine the cost-efficiency of the mobile dental clinic utilized in the West Rand region over the first year of implementation	An accessible and cost-efficient service, with a cost-efficiency ratio of 1.636 (63.6%), yielding a net margin ratio of 0.3889, was provided. The services, including tooth extraction, restoration, oral hygiene procedure and fissure sealants provided by dentists, dental therapists, and oral hygienists using mobile dental clinic.	The Mobile Oral Health Care System is an accessible and cost-efficient service was provided to health districts in the West Rand region.
Bhayat and Chikte, 2018 (76)	South Africa	Descriptive analysis: A review of the current disease burden based on local epidemiological studies and the number of oral health personnel registered with the Health Professions Council of South Africa	Oral health services are rendered by oral hygienists, dental therapists, dentists, and dental specialists. Dental caries remains one of the most prevalent conditions, and much of them are untreated. The majority of oral care providers are employed in the private sector even though the majority of the population access the public sector which only offers a basic package of oral care	Infrastructure at primary health care facilities needs to be improved to improve the effective utilization of dentists working in the community. More mid-level workers’ need to be trained at the academic training institutions.
Nkambule et al., 2022 (45)	South Africa	Descriptive analysis: To analyze the five National Health Goals for 2030 with reference to the impact they could have on dentistry in South Africa	Some of the goals of the 2030 Human Resources for Health Strategy: Investing in the Health Workforce for Universal Health Coverage” are being attained	The public sector must create more posts, appoint community dentistry specialists to managerial roles, upskill current managers, increase mid-level worker positions (oral hygienists and dental therapists), and improve mid-level workers’ financial packages. Tertiary institutions should align training with oral disease burdens, establish career pathways for mid-level workers, and support oral health research and manager training
El Tantawi et al., 2020 (41)	Egypt	Ecological study: assessed dentist availability in Egypt including (1) changes over 20 years; (2) spatial distribution; and (3) association with supply, potential demand for care and economic conditions	Dentists per 1,000 population were randomly distributed over the country and the ratio reached 0.18 in 2014, indicating a shortage despite the increasing number of dental graduates since 1995 (667.1%). Previous dentist availability (η2 = 0.60) and increase in wages (η2 = 0.48) had the greatest impact on dentist availability.	Egypt faces a problem of dentist shortage that has not been offset by the increase in dental graduates. Improving the economic conditions and incorporating health care into the national development plan may improve the situation
Amedari et al., 2022 (46)	Nigeria	Scoping review: to describe the building blocks of the oral health system, including the role that the community plays in strengthening the oral health system in Nigeria	Increasing dental clinics is essential to enhance access to oral health services. Expanding the dental workforce through training junior cadre professionals and involving community health practitioners for basic oral care is critical. Regulating access to dental medications and including oral disease treatments in national guidelines will standardize care. Greater government health spending, with increased allocation for oral health, is urgently needed. Effective national policies, coupled with community participation, engagement, and ownership, are key to achieving oral health system goals	Strengthening Nigeria’s oral health system requires urgent action on all building blocks and cross-cutting interventions, with a strong emphasis on community involvement to sustain organizational change
Adeniyi et al., 2012 (47)	Nigeria	Descriptive analysis: to assess the strengths and weaknesses of the oral health care system in Nigeria and to outline broad policy options for strengthening the system	There has been some progress in the oral healthcare system in Nigeria, but the system still lacks many essential qualities. It remains neither effective nor efficient, with available resources being severely limited and stretched thin across various areas. The system is unresponsive to the needs of the population, and there is a noticeable lack of proper stewardship in managing it	Secure adequate resources for the immediate implementation of the national oral health policy. Increased research on oral health issues is also essential. Coordination of all sector stakeholders is needed for success.

An analysis of studies on oral health systems and workforce in Africa reveals a complex interplay of structural, financial, and educational challenges. These include systemic neglect, educational barriers, and financial constraints, disproportionately affecting vulnerable groups such as children and low-income populations (34).

The findings highlight the severe shortage of oral health professionals, exemplified by a dentist-to-population ratio of 0.44 per 10,000 in the WHO African Region, reflecting maldistribution and the low priority given to oral health (35–37). Libya, for example, relies heavily on general dental practitioners, with significant shortages of dental technicians and nurses, compounded by an absence of clear policies (34). Similarly, Mozambique has only 72 dentists, mostly concentrated in urban areas, highlighting workforce maldistribution (36) as also observed in Nigeria (37). Despite training programs, rural areas remain underserved, as evidenced across several countries, including Benin and Congo (36).

Efforts to strengthen dental education demonstrate promise. Rwanda’s first dental school, in partnership with international institutions, developed tools to improve curriculum delivery and resource management, aligning with the Human Resources for Health program (38, 39). Similarly, Mozambique’s localized training initiatives have enhanced the capacity of dental therapists and assistants, emphasizing leadership development (40). However, Egypt’s dentist shortage persists despite a 667% increase in dental graduates over two decades, underlining the need for economic reforms and better spatial distribution (41).

Oral health service provision remains limited, with public sectors in countries like Libya offering only emergency care and extractions, reflecting broader systemic neglect (34). In South Africa, mobile dental units have emerged as an accessible and cost-efficient alternative, providing both curative and preventive care while serving as training platforms for dental students (42, 43). Economic evaluations suggest that transitioning to a dental therapy-led model could further reduce costs while maintaining service quality (43). Similar recommendation for workforce transition was made for Nigeria (37).

Strengthening oral health systems across Africa requires a multifaceted approach to address workforce challenges such as low density, uneven distribution, shortages in specialized roles, projected shortfalls, and reliance on the private sector. In addition, key issues such as the poor integration of oral health into broader healthcare services, inadequate oral healthcare infrastructure, gaps in education and training of dentists and mid-level manpower, oral health policy and financial deficiencies, limited oral health preventive programs, and public-private inequities where underfunded public services provide only basic care while the private sector dominates comprehensive service delivery all need to be addressed.

Efforts to strengthen the oral health system should include expanding dental education programs, promoting task-shifting through mid-level workers like dental therapists, and incentivizing professionals to serve in rural and underserved areas (37, 44, 45). Scaling up mobile dental units, particularly for school-based programs, could significantly enhance accessibility and cost-efficiency (43). Collaborative frameworks, such as partnerships between institutions (38), and community participation, engagement, and ownership (46) should be leveraged to optimize training, oral health reach, and resource-sharing. Dental education systems also need reforms to expand and include mid-level workers, aligning training with regional oral disease burdens, and admitting minority populations into the dental training institutions to improve culturally sensitive care provision (44–47). Renumeration packages need to be improved to enhance workforce retention and service efficiency (37, 45). These findings emphasize the need for context-specific, innovative strategies to strengthen oral health systems, address workforce gaps, and ensure equitable access to care across Africa (34, 35, 45).

Integrating oral health into national health policies is essential to ensure dedicated funding, address rural–urban workforce disparities, improve primary oral healthcare infrastructure (37, 45, 46), and to alignment with broader primary healthcare objectives. Furthermore, developing contingency plans for oral health service delivery in fragile and conflict-affected settings is necessary to maintain service continuity in these contexts. Policies must include plans for robust systems to track workforce distribution, service delivery outcomes, and policy impacts should be implemented to monitor progress and inform improvements. Lastly, promoting evidence-based decision-making through regular assessments and active stakeholder engagement will ensure that policies are responsive to evolving needs and challenges in the oral health landscape.

## Africa CDC’s role in strengthening integrated oral health systems in Africa

The Africa CDC, established by the African Union in 2016 and launched officially in 2017, was created to support African Union member states in tackling public health challenges (48). It serves as a vital platform where member states can collaborate, exchange insights, and share strategies from public health interventions (49), thus positioning itself as a driving force for public health innovation beyond mere disaster management. Africa CDC’s mandate emphasizes disease prevention, health system strengthening, and promoting health security, positioning it well to elevate oral healthcare within a broader, integrated healthcare framework across the continent.

Oral health is often overlooked in public health policies, yet it intersects significantly with other NCDs and infectious diseases, making it an essential component of comprehensive health strategies. Africa CDC’s broader goal of addressing non-communicable diseases and infectious diseases provides a solid foundation for including oral health within its programs. By treating oral health as a part of overall health security and disease prevention, Africa CDC can better respond to the continent’s oral health problems, which contribute heavily to morbidity and affect quality of life across all age groups.

Africa CDC has demonstrated an impressive capacity for uniting African nations to respond to major public health threats, including the COVID-19 pandemic and mpox outbreaks. During the COVID-19 pandemic, Africa CDC played a key role in mobilizing resources, guiding member states on best practices, and coordinating continent-wide testing and vaccination efforts (50). This successful model of rapid mobilization, resource-sharing, and consistent guidance greatly minimized the pandemic’s spread and impact across the continent. Similarly, in response to the mpox outbreak, Africa CDC enabled rapid information-sharing, bolstered laboratory capacities, and supported the formation of national response plans (51, 52). Its coordinated strategies have not only safeguarded public health but have also demonstrated Africa CDC’s effectiveness in unifying countries in Africa toward common health goals.

Building on this established framework, Africa CDC can extend its role to address oral health challenges. This would involve strengthening existing health infrastructures and partnerships to improve oral healthcare prioritization, accessibility, and quality. By integrating oral health within its broader health programs, Africa CDC can support preventive, diagnostic, and therapeutic interventions at the community level. Furthermore, just as it facilitated national responses for pandemic control, Africa CDC can develop oral health policies and guidelines that can be adopted by member states to improve preventive and curative care at primary healthcare facilities. This way, the institution would be strengthening integrated systems for the prevention and control of the high oral health disease burden on the continent in line with its 2023–2027 strategic plan (53).

Africa CDC can strengthen its oral health response by adapting the US CDC’s global health security model within the WHO Health Systems Strengthening Framework, which offers a comprehensive structure based on six building blocks: service delivery, workforce, information systems, essential medicines, financing, and governance (54). This approach would allow Africa CDC to apply the US CDC’s goals—stopping health threats at their source, leveraging data for prevention, and promoting health equity—to improve oral health outcomes in a coordinated, sustainable, and equitable way across the continent.

**Strengthening surveillance and data-driven policy development**: Africa CDC’s mandate includes disease surveillance, which is crucial for understanding and addressing the oral health landscape. By incorporating oral health indicators into existing surveillance systems, the Africa CDC can monitor disease trends, measure intervention effectiveness, and guide evidence-based policy development. Reliable data on oral health can also help identify disparities across regions, track progress, and ensure that resources are allocated equitably. To address threats at their source, Africa CDC can also integrate oral health assessments into primary care and public health surveillance systems, enabling early identification and intervention. This would support tracking high-risk populations and monitoring the prevalence of diseases like dental caries and oral cancers.

In alignment with its role in coordinating data-driven policies, the Africa CDC can support member states in establishing robust oral health monitoring frameworks that integrate with other NCD data collection efforts, fostering a comprehensive approach to public health.

Data collection and analysis are essential to Africa CDC’s strategy for prevention. By establishing a continental database on oral health metrics, Africa CDC can provide valuable insights into disease prevalence, risk factors, and intervention outcomes across different regions. These data-driven insights allow member states to allocate resources more effectively, tailor interventions to specific population needs, and anticipate challenges. In turn, this data-centric approach would improve health outcomes and maximize the impact of investments in health infrastructure. This process can be jointly managed with other stakeholders like WHO Africa Region.

By emphasizing the need for integrated health policies that address oral health, the Africa CDC can drive continental and national commitments that prioritize oral health alongside other critical health issues. Additionally, as part of its mandate to improve health systems, the Africa CDC can push for policies that integrate oral health into primary healthcare (PHC), making essential services more accessible to underserved populations.

**Providing technical assistance for health system integration**: The Africa CDC’s mandate encompasses providing technical assistance to member states to enhance health service delivery. Through its coordinated, data-driven approach, Africa CDC has the potential to lead a transformative shift toward integrated health systems in Africa, where oral health is recognized as a vital component of overall well-being and health equity across the continent.

To integrate oral health within PHC and Universal Health Coverage frameworks, the Africa CDC can assist countries in developing national guidelines, best practices, and service standards for oral health. Technical support can also cover areas such as resource allocation, workflow integration, and establishing referral systems, helping countries streamline oral health services within existing health systems. Through technical assistance, the Africa CDC can facilitate the adaptation of oral health interventions to diverse healthcare settings, ensuring that these services meet the specific needs of African populations.

Africa CDC is uniquely positioned to address the integration challenges that many African countries face in their health systems. While multiple countries in Africa have attempted to facilitate integration across healthcare sectors, results have often been limited (55) due to fragmented systems, inconsistent policy implementation, and resource constraints (56, 57). Africa CDC can make a meaningful difference by acting as a central, coordinating body that supports a unified approach across the continent, drawing on its mandate to improve health security, health system resilience, and disease prevention capabilities for the African Union member states.

A key strength of Africa CDC lies in its ability to bring together expertise, establish evidence-based guidelines, and foster partnerships across regions and sectors. By creating standardized protocols and facilitating knowledge exchange among member states, Africa CDC can help address inconsistencies in policy and practice that often hinder integration efforts. For example, Africa CDC can develop shared frameworks for integrating oral health within primary health systems, ensuring that oral health is prioritized alongside other health services rather than treated as a separate silo. This would directly address gaps in service delivery, particularly in rural and underserved communities, where access to specialized oral health services remains limited.

Furthermore, Africa CDC’s approach to integration can build on its experience in coordinating responses to public health crises, such as the COVID-19 pandemic and mpox outbreaks. During these crises, Africa CDC effectively mobilized resources, unified data collection, and supported member states in adopting cohesive response measures (52). This experience can be adapted to strengthen integrated health systems in non-crisis settings, helping ensure that countries are better equipped to provide comprehensive, continuous care that includes preventive and curative oral health services.

The Africa CDC’s ability to leverage partnerships and mobilize resources is another key factor in advancing health system integration. Collaborating with international health organizations, government bodies, and the private sector enables Africa CDC to pool resources, share technology, and increase the availability of essential medicines and supplies across the continent. For example, partnerships with dental product manufacturers could ensure a stable supply of preventive materials, such as fluoride toothpaste and dental sealants, even in low-resource settings.

Finally, the Africa CDC can promote sustainable health financing models that include oral health within broader health agendas, advocating for national budgets and policies that make integrated health services financially viable. By providing technical guidance on financing mechanisms and supporting countries in designing Universal Health Coverage policies that incorporate oral health, Africa CDC can address the financial barriers that have previously hindered integration efforts.

**Capacity building for oral health workforce development**: One of the Africa CDC’s key responsibilities is to strengthen human resource capacity for health. By developing training programs and capacity-building initiatives that include oral health, the Africa CDC can address the shortage of oral health professionals across the continent. Training for general health workers, such as nurses and PHC providers, on basic oral health skills can enhance task-shifting and the integration of oral care within routine healthcare services.

Furthermore, Africa CDC’s mandate to promote resilience in health systems aligns with developing a versatile, community-oriented workforce capable of delivering preventive and primary oral health services, particularly in underserved areas. The Africa CDC is championing efforts at strengthening primary healthcare delivery system through the integration of community health workers into the formal healthcare systems in Africa, and ensuring they are adequately trained, supervised, and compensated (58). To this effect, Africa CDC secured a $900 million commitment to community health investments (59).

Unfortunately, there are currently no plans to integrate oral health promotion and prevention into the activities of the community health workers. Yet, there are suggestive evidence that community health workers can play roles in supporting oral health in Africa by educating communities on oral hygiene, promoting regular dental check-ups, and providing preventive services like fluoride application and dietary counseling to reduce sugar intake. They can identify early signs of oral diseases, refer patients for further treatment, and help prevent the progression of serious conditions (60).

Building the capacity of this cadre of oral health care worker can contribute to reducing the oral health inequality resulting from shortage of the oral workforce on the continent. This strategic initiative can take several forms, including developing comprehensive training programs for oral health care workers to improve both culturally appropriate theoretical knowledge and practical skills to undertake health education campaigns and screenings in schools or community centers. In addition to initial training, ongoing professional development is crucial for keeping oral health care workers updated on the latest practices, leveraging on technologies, research in the field, and to promote collaboration with other health professionals to provide comprehensive care. National policies are, however, needed to create a favorable environment for recruiting and retaining community health workers to provide oral health professionals, particularly in underserved areas.

**Promoting preventive and community-based oral health interventions**: Prevention is a central focus of the Africa CDC’s mandate, and it is crucial for reducing the incidence and severity of oral diseases. By advocating for preventive strategies, the Africa CDC can support countries in launching community-based programs that raise awareness about oral hygiene, healthy diets, and regular dental check-ups using mobile dental health clinics where feasible. Public health campaigns tailored to the social and cultural contexts of African communities can empower individuals to prioritize oral health, reducing the need for advanced treatments.

The Africa CDC can significantly improve access to oral health services in Africa by leveraging the Community-Led Monitoring (CLM) program, particularly through Global Fund initiatives to integrate CLM into national health systems (61). This grassroots approach empowers communities to monitor their oral health needs, identify service gaps, and advocate for better access to care. By enhancing data collection on oral health challenges, CLM enables targeted interventions and informed resource allocation. It also improves service delivery by providing real-time feedback on deficiencies and fostering accountability among providers. In addition, CLM supports health education initiatives, facilitates access to essential medicines, and empowers communities to advocate for policies that prioritize oral health. By addressing social determinants of health and strengthening partnerships among stakeholders, the Africa CDC can tackle oral health disparities and ultimately promote better health outcomes and equity across the continent.

The Africa CDC can also promote school-based programs that encourage children to adopt oral health practices early on, contributing to lifelong oral health and overall well-being. Integrating oral health education into existing school programs for mpox control offers the Africa CDC a strategic opportunity to enhance children’s health outcomes. Schools are pivotal in reaching children, who can become lifelong advocates for healthy practices. School-based programs can also help normalize discussions about oral health and reduce stigma associated with oral diseases. In addition, schools can serve as effective distribution points for oral health resources, such as toothbrushes and educational materials, particularly in underserved communities. Moreover, embedding oral health components into current health programs addressing infectious diseases like mpox fosters a holistic view of health, highlighting the links between oral hygiene and infection prevention. This would require advocating for policies that promote oral health within national school health frameworks that ensures that oral health education becomes an integral part of school curricula.

**Facilitating cross-border collaboration and knowledge sharing**: In line with its mandate to foster collaboration among AU member states, the Africa CDC can serve as a regional hub for sharing best practices, research findings, and innovative strategies for oral health integration. Africa CDC currently serves as a regional hub for public health in Africa, playing a critical role in coordinating health initiatives and responses across the continent (62). By acting as a central resource for member states, the Africa CDC can facilitate collaboration and partnerships with governments, non-governmental organizations, and international agencies and information sharing, enhance capacity building, and supports the implementation of oral health strategies tailored to the unique needs of African populations.

By facilitating cross-border collaboration, the Africa CDC can help countries address shared challenges, such as workforce shortages and limited resources, through coordinated efforts. Through workshops, conferences, and knowledge-sharing platforms, the Africa CDC can foster a culture of collective learning, enabling countries to adopt successful models and approaches from one another. This network of cooperation can contribute to harmonized oral health strategies that strengthen healthcare resilience across Africa.

**Mobilizing resources and funding for oral health programs**: Resource mobilization is integral to the Africa CDC’s mandate. The Africa CDC can play a pivotal role in attracting funding for oral health initiatives by demonstrating their relevance to overall health security and economic development. By engaging with international organizations, donors, and private sector stakeholders, the Africa CDC can advocate for increased investment in oral health infrastructure, workforce training, and community-based prevention programs. In addition, by presenting oral health as a component of the broader non-communicable diseases’ agenda, the Africa CDC can tap into existing funding channels dedicated to non-communicable diseases prevention and control, promoting a sustainable funding model for oral health programs. This resource mobilization should be driven by the development of oral health policies and annual costed workplans.

**Local production of oral health products**: In addition, Africa CDC’s local production agenda is central to advancing health independence in Africa by building the capacity to produce essential dental products and resources on the continent. This strategy focuses on reducing reliance on international imports for medicines, vaccines, medical equipment, and consumables, which often leads to supply shortages, higher costs, and delayed access (63). For oral health, the local production agenda is particularly relevant, as it can improve the availability and affordability of essential oral health supplies, including dental equipment, fluoride-based products, and antibiotics for dental infections. Local production can bridge critical gaps in oral health disparities associated with poor access to affordable, quality care, and support a sustainable health ecosystem that can better withstand global market fluctuations.

## Challenges the Africa CDC may face in promoting oral health

The Africa CDC plays a pivotal role in strengthening health systems, disease prevention, and emergency response across the continent (64), but several human resources, financial constraints, and infrastructure deficiencies hinder its ability to effectively address these health challenges in Africa (65). These challenges are particularly impactful in advancing oral health initiatives across the region, and can hinder the effective promotion and integration of oral health and integrate into broader public health strategies.

Workforce shortages, particularly in specialized fields like dental public health, epidemiology, and laboratory sciences, can limit the Africa CDC’s ability to address oral health needs. The continent suffers from a shortage of trained oral health professionals, including dental hygienists, specialists, and public health experts (35), who can drive national and regional oral health initiatives, such as preventive education, screening, and treatment programs. In addition, the brain drain, where highly trained professionals leave Africa for better opportunities abroad (66), exacerbates this issue, depriving the Africa CDC of skilled personnel crucial for advancing oral health on the continent. Furthermore, limited training and capacity-building opportunities for health professionals in Africa result in a workforce that may not be fully equipped to address emerging public health challenges, including the growing burden of oral diseases (67).

In addition, financial challenges are a major obstacle to the Africa CDC’s capacity to integrate oral health into broader public health strategies. Insufficient funding hampers the development of national oral health programs, restricts the provision of necessary resources for oral health initiatives, and delays critical interventions. The reliance on external donors to fund health (68) also means that oral health programs may be fragmented and misaligned with the priorities of local communities. Moreover, inefficient resource allocation—due to limited financial management capabilities—often leads to suboptimal utilization of available funds (69), undermining the potential for scaling up successful oral health interventions. The lack of sustainable financing for oral health initiatives can limit Africa CDC’s capacity to implement long-term strategies for improving oral health across the continent.

Inadequate infrastructure remains a significant challenge. Many African countries lack the necessary capacity and equipment to support comprehensive oral health surveillance, including the detection and monitoring of oral diseases. This infrastructure gap would hamper the Africa CDC’s ability to track and respond effectively to the escalating oral health crisis, as seen in the ongoing mpox outbreaks (70). Weak health information systems would further complicate efforts to collect and analyze oral health data, making it difficult to identify trends, allocate resources, and inform policy (71). The absence of robust data—referred to as health data poverty (72)—poses a major obstacle to advocating for and designing evidence-based interventions (73). In addition, logistical challenges, such as unreliable transportation networks, can disrupt the delivery of essential oral health supplies, including fluoride varnishes and dental equipment, to remote and underserved areas.

More importantly, the systems and structures to integrate oral health into ongoing Africa CDC’s ongoing operations, needs to be created. The African CDC needs to create dedicated structures, such as specialized units, to link oral health with broader public health programs like non-communicable diseases, maternal health, and infectious disease control. Collaboration with WHO AFRO and other stakeholders would be essential to create and ecosystem to play this crucial role.

## Conclusion

Incorporating oral health into Africa CDC’s framework for disease prevention and health security could drive substantial improvements in public health outcomes. The new public health order championed by the Africa CDC, can serve as a framework to enable member states and public health leaders and institutions across the continent to collaborate, align efforts, and optimize national and regional public health resources to collectively ensure Africa’s oral health security. This would require integrating oral health into routine disease surveillance, strengthening data on oral disease burdens, promoting oral health education campaigns and prevention, and increasing accessibility to oral health services in underserved regions. Furthermore, by coordinating with member states to include oral health within Universal Health Coverage and non-communicable diseases frameworks, Africa CDC can ensure that oral health receives adequate attention within healthcare policies and budgets. This would lead to a more holistic, health security-oriented approach that incorporates oral health as an essential component of African health resilience. In addition, by advocating for oral health prioritization in national health agendas, Africa CDC can drive the development of a continental strategy aligned with the African Union’s Agenda 2063 of building resilient oral health systems across member states.

## Data Availability

The original contributions presented in the study are included in the article/supplementary material, further inquiries can be directed to the corresponding author/s.
